# Awareness and acceptability of herpes zoster vaccination in people living with HIV^☆^

**DOI:** 10.1016/j.pmedr.2025.103143

**Published:** 2025-06-16

**Authors:** Christian Motet, Agnès Libois, Charlotte Martin, Nicolas Dauby

**Affiliations:** a Department of Infectious Diseases, CHU Saint-Pierre, Université Libre de Bruxelles (ULB), Brussels, Belgium; b School of Public Health, Université Libre de Bruxelles (ULB), Brussels, Belgium; c U-CRI, Université Libre de Bruxelles (ULB), Brussels, Belgium

**Keywords:** Herpes zoster, Immunization, HIV, Vaccine acceptance, RZV

## Abstract

**Objective:**

The recombinant herpes zoster vaccine is recommended for aging populations to prevent herpes zoster. We aimed to assess awareness of herpes zoster and acceptance of the herpes zoster vaccine acceptance among people living with HIV (PLWH).

**Methods:**

We conducted a written survey among PLWH attending an HIV outpatient clinic in Brussels from March 2022 to April 2023. Participation was voluntary. We collected epidemiological data, assessed knowledge of herpes zoster and opinion about herpes zoster vaccine and vaccination in general. We described the surveyed cohort and reported their attitudes toward vaccination. Herpes zoster vaccine acceptance and general vaccine acceptance were defined as two binary outcome variables. We performed binary logistic regression to identify factors associated with vaccine acceptance.

**Results:**

A total of 327 patients completed the questionnaire. While 64 % reported being aware of herpes zoster, only one third reported a link between herpes zoster and varicella infection. The majority (77.1 %) declared being in favour of overall vaccination. Willingness to be vaccinated (regardless of vaccine type) was the factor associated with herpes zoster vaccine acceptance. General vaccine acceptance was associated with the belief that vaccination is useful for preventing infectious diseases.

**Conclusions:**

Knowledge and awareness about herpes zoster were low among PLWH surveyed. Herpes zoster vaccine acceptance was more related to general attitudes toward vaccination rather than specific knowledge about herpes zoster.

## Highlights

• A survey among people living with HIV on herpes zoster and vaccination.
• Awareness about herpes zoster in people living with HIV is low.
• The majority of the respondents favors vaccination in general.
• Herpes zoster vaccine acceptance relates more to vaccine attitudes than knowledge.

## 1 Introduction

Herpes zoster results from the reactivation of varicella zoster virus (VZV) and usually presents as painful vesicular lesions of the skin and is complicated by chronic pain known as post-herpetic neuralgia (PHN) in about 15 % of cases (Gershon et al., 2015). Both acute herpes zoster and PHN impact significantly the quality of life affecting sleep quality, relationships with others and enjoyment of life (Drolet et al., 2010). While antiretroviral therapy has been associated with a significant decrease of herpes zoster incidence among people living with HIV (PLWH) (Moanna and Rimland, 2013), epidemiological studies in Europe and North America still indicate an increased risk of herpes zoster and PHN among PLWH. Moreover, aging PLWH now face an increasing risk of herpes zoster associated with aging, similarly to the general population (Dauby et al., 2023).

The recombinant herpes zoster vaccine (RZV), based on VZV glycoprotein E and ASO1 adjuvant system, has demonstrated great efficacy in the prevention of both herpes zoster (97.2 % in adults aged 50 or older after 3.5 years, 89.8 % in adults aged 70 or older after 3.2 years) and PHN (91.2 % in adults aged 70 or older after 3.8 years) (Lal et al., 2015; Cunningham et al., 2016) and is now recommended for PLWH according to European and North-American guidelines (Thompson et al., 2021; EACS Guidelines, 2023). PLWH in Belgium (from 16 years old) are recommended to get RZV and which is accessible through reimbursement by social security (Vaccination contre l'herpès zoster. Conseil Supérieur de la Santé, 2025; Remboursement. CBIP, 2025).

Vaccine uptake may be impacted by vaccine hesitancy, a concept that includes the different hurdles to vaccine acceptance. Vaccine hesitancy may be in part related to lack of awareness (complacency), or reduced confidence in vaccines or specific vaccine (MacDonald, 2015). Although different studies have been performed among the general population, no study has specifically assessed herpes zoster vaccine awareness and acceptability among PLWH.

In this study we aimed to assess acceptance of overall vaccination, herpes zoster vaccine and the knowledge about herpes zoster in outpatient PLWH followed in an HIV center in Brussels, Belgium.

## 2 Methods

We conducted a cross sectional study to explore herpes zoster awareness, herpes zoster vaccine acceptance and general vaccine acceptance among PLWH through a survey. The study population consisted of outpatients living with HIV attending the HIV clinic reference center at CHU Saint-Pierre, Brussels, Belgium. While waiting to be seen by a doctor, patients were invited to take part in a voluntary written survey. This involved completing a 29-question paper survey (**Appendix 1**) based from an another similar study performed in the French elderly population (Del Signore et al., 2020). The questionnaire was translated into seven languages (French, Dutch, English, Spanish, Portuguese, Polish and Romanian) to improve accessibility. When the patients considered they had completed the questionnaire, they gave it to the consulting physician. The study was carried out from March 2022 to April 2023. To prevent potential duplicate entries, we excluded any subsequent surveys completed by the same patient.

Through the questionnaire and our HIV patient database we collected epidemiological data (age, sex, birth country, level of education, sexual orientation). A self-reported brief medical history (chronic diseases and personal history of herpes zoster) was also collected via the questionnaire. The survey aimed to obtain patient's awareness about herpes zoster, opinion about vaccines and opinion about getting vaccinated against herpes zoster if they had risk factors. All data were collected using REDCap forms hosted at CHU Saint-Pierre (Harris et al., 2009). The study met the institution's guidelines for protection of human subjects concerning safety and privacy and was approved by the CHU Saint-Pierre Ethic Committee (CE/22–01-26). The confidentiality of participants' data was preserved in accordance with the General Data Protection Regulation.

### 2.1 Descriptive data

We first described the surveyed cohort and its opinion regarding vaccination (herpes zoster vaccination and general vaccination).

### 2.2 Studied outcomes and factors associated

We defined two binary outcome variables: herpes zoster vaccine acceptance and general vaccine acceptance. Herpes zoster vaccine acceptance was analyzed in relation to epidemiological data, medical history, awareness and knowledge about herpes zoster, and attitudes toward general vaccination. General vaccine acceptance was assessed based on epidemiological data, medical history and the patient's perception of the effectiveness of vaccination as a preventive measure for infectious diseases.

### 2.3 Statistical analysis

Categorical variables were presented as percentages while continuous variables were presented with means and standard deviation. To identify predictors of vaccine acceptance or refusal, we performed multivariable logistic regression using backward stepwise selection. Variables were excluded from the resulting model if *p*-value >0.10. Model fit was assessed using Chi-square goodness-of-fit tests and deviance, Nagelkerke's pseudo-R^2^, and the area under the ROC curve (AUC). Data analysis was performed with IBM SPSS 28 (IBM Corp. 2021, Armonk, NY).

## 3 Results

### 3.1 Population characteristics

A total of 327 PLWH completed the survey. Population characteristics are summarized in Table 1. The mean age was 49 years, and the majority was male (239/327, 73.1 %), from Caucasian ancestry (201/327, 61.5 %) and were men having sex with men (MSM) (173/298, 58.1 %). About a third were from Sub-Saharan Africa ancestry. These characteristics were similar to that of the PLWH in Belgium (Deblonde et al., 2022). Slightly over half of patients reported having completed graduate studies. Additionnaly, 77 (23.8 %) patients reported one or several other medical condition(s) than infection with HIV with diabetes and cardiovascular diseases being the most common. Most of the patients declared being in favour of vaccination (77.1 %) in general and considered vaccination a good tool for preventing infectious diseases (84.5 %). A slight majority (63.5 %) of the patients agreed to be vaccinated if recommended for any vaccine. Finally, a high proportion of the patients (90.8 %) reported being vaccinated against COVID-19.

**Table 1:** Characteristics of patients living with HIV who participated to the survey, CHU Saint-Pierre outpatient HIV center, Brussels – Belgium (2022−2023).

Description	n (%)
Mean age (years) +/− SD	49.0 (+/− 12.2)
Male	239 (73.1)
*Ethnicity*	
– Caucasian	201 (61.5)
– Sub-Saharan African	101 (30.9)
– Asian	9 (2.8)
– Other	14 (4.3)
– Unknown	2 (0.6)
*Sexual orientation*	
– MSM	173/298 (58.1)
*Education level (maximum)*	
– Graduate studies	171/322 (53.1)
*Underlying chronic disease*	77/323 (23.8)
– Diabetes	27/323 (8.4)
– Cardiovascular disease	30/323 (9.3)
– Pulmonary disease	12/323 (3.7)
– Renal disease	7/323 (2.2)
– Cancer	12/323 (3.7)
*Knowledge, personal history of herpes zoster and vaccine acceptance*	
People who know herpes zoster	205/320 (64.1)
People who know that varicella and herpes zoster are linked	113/320 (35.3)
Personal history of varicella	195/323 (60.4)
Personal history of herpes zoster	80/315 (25.4)
– People who received antiviral treatment for herpes zoster	45/80 (56.3)
– Acute pain	64/80 (80.0)
– Chronic pain	32/80 (40.0)
People who think that herpes zoster causes pain	169 (51.7)
Type of pain as reported by patients who believe that herpes zoster is painful	
– Acute pain	32/169 (18.9)
– Chronic pain	25/169 (14.8)
– Acute and chronic pain	59/169 (34.9)
– Do not know	53/169 (31.4)
People who know a relative with a history of herpes zoster	109/269 (40.5)
People who think herpes zoster is severe	155 (47.4)
People who are aware of the herpes zoster vaccine	42 (12.8)
Herpes zoster vaccination: people who are willing to get vaccinated	201/326 (61.7)
*General vaccine acceptance*	
Regarding vaccination	
– Strongly in favour	146/305 (47.9)
– In favour	89/305 (29.2)
– Neutral	57/305 (18.7)
– Against	11/305 (3.6)
– Strongly against	2/305 (0.7)
Willing to be vaccinated if indicated (any vaccine)	198/312 (63.5)
About vaccination as a prevention method against infectious diseases	
– People who think vaccination is a good prevention method against infectious diseases	267/316 (84.5)
Vaccination against COVID-19	
– People vaccinated against COVID-19	287/316 (90.8)

MSM: men having sex with men, SD: standard deviation.

### 3.2 Herpes zoster awareness and history of disease

Most patients (64.1 %) reported knowledge about herpes zoster but only one third were aware of the link between varicella and herpes zoster. One quarter (25.4 %) reported a personal history of herpes zoster, and among these, 32 of them (40 %) experienced chronic pain consistent with PHN. Of note, 40.5 % of respondents knew a relative who had had herpes zoster. Only a minority (12.8 %) reported being aware of the herpes zoster vaccine.

### 3.3 Herpes zoster vaccine acceptability

Regarding herpes zoster vaccination, 201 (61.7 %) individuals indicated they would agree to be vaccinated if they had risk factors. To assess herpes zoster vaccine acceptability, we compared patients who would accept herpes zoster vaccine to those who would not accept or answers “I don't know” (Table 2).

**Table 2:** Determinants of herpes zoster vaccine acceptance in case of risk factors among people living with HIV, CHU Saint-Pierre outpatient HIV center, Brussels – Belgium (2022–2023) – Multivariable Analysis Results.

	Agree – n (%)	Do not agree/do not know – n (%)				
Variables included in initial model			Final model variables			
	*n* = 201	n = 126	β	SE	OR	95 %CI
Mean age (years)	48.3	50.1				
Male	160 (79.6)	79 (62.7)				
*Education level*						
– Graduate studies	116/198 (58.6)	55/124 (44.4)				
– Secondary/primary school or unschooled	82/198 (41.4)	69/124 (55.6)				
*Ethnicity*						
– Sub-Saharan African	55 (27.4)	46/124 (37.1)				
– Non Sub-Saharan African	146 (72.6)	78/124 (62.9)				
MSM	120/145 (82.8)	53/66 (80.3)				
Underlying chronic disease	54/199 (27.1)	23/124 (18.5)				
Personal history of varicella	125 (62.2)	70 (55.6)				
Personal history of herpes zoster	57/193 (29.5)	23/122 (18.9)				
Patients who know what herpes zoster is	135/199 (67.8)	70/121 (57.9)				
People who know a relative with a history of herpes zoster	73/166 (44.0)	36/103 (35.0)				
People who know herpes zoster vaccine	29 (14.4)	13 (10.3)				
People who think vaccination is a good prevention tool against infectious diseases	181/195 (92.8)	86/121 (71.1)				
People who are in favour of vaccination	173/192 (90.1)	62/113 (54.9)				
Willing to be vaccinated if indicated (any vaccine)	176/193 (91.2)	22/119 (18.5)	4.1	0.5	57.7	19.9–167.6
People who are vaccinated against COVID-19	189/195 (96.9)	98/121 (81.0)				

Stepwise backward method was used. Variables eliminated when p-value >0,10. Model fit statistics: Χ^2^ (1) = 84.2 p-value <0.001; pseudo-R^2^ by Nagelkerke = 0.6; AUC (ROC): 0.8.

β: β coefficient, CI: confidence interval, MSM: men having sex with men, OR: odds ratio, SE: standard error for β coefficient.

The proportion of women and patients from Sub-Saharan Africa were significantly higher in the group of patients who did not agree to get vaccinated against herpes zoster in the presence of risk factors. In the group of patients who agreed to get vaccinated, there were higher proportion of patients who completed graduate studies. Patients who were aware of herpes zoster and those with a personal history of herpes zoster were also more likely to accept vaccination. Almost all patients who agreed to receive the herpes zoster vaccine were in favour of vaccination in general, would accept any indicated vaccine and reported being vaccinated against COVID-19. Age was assessed and found to have no significant influence on herpes zoster vaccine acceptance (**Appendix 2**). Among the individuals who refused or were hesitant to receive herpes zoster vaccine (*n* = 126), the most commonly cited reasons were fear of long term side effects (30.2 %), the non-mandatory nature of the vaccine (19.8 %), fear of local side effects (10.3 %) and cost (4.0 %).

After multivariable analysis, the only variable associated with herpes zoster vaccine acceptability was willingness to be vaccinated if indicated for any vaccine (OR 57.7, 95 % CI [19.9–167.6]) (Table 2).

### 3.4 Predictors of general vaccination acceptance

We identified factors associated with general vaccine acceptance by comparing individuals who were in favour of vaccination to those who were against or unsure. A higher proportion of women and patients of Sub-Saharan African ancestry were found among those opposed to or hesitant about vaccination. The pro-vaccination group included a greater proportion of individuals who had completed graduate studies. Nearly all patients in favour of vaccination had been vaccinated against COVID-19 and believed that vaccination is an effective tool for preventing infectious diseases (Table 3).

**Table 3:** Predictors of general vaccination acceptance in people living with HIV, CHU Saint-Pierre outpatient HIV center, Brussels – Belgium (2022–2023) – multivariable analysis results.

	Favors vaccination – n (%)	Against vaccination/do not know – n (%)				
Variables included in initial model			Final model variables			
	*n* = 235	*n* = 70	β	SE	OR	95 %CI
Age (mean)	49.1	49.0				
Male	187 (79.6)	39 (55.7)				
Sub-Saharan African	59/234 (25.2)	31/69 (44.9)				
MSM	141/170 (82.9)	27/32 (84.4)				
Graduate studies	139/231 (60.2)	29/69 (42.0)				
Underlying chronic disease	55/232 (23.7)	18 (25.7)				
People who think vaccination is a good prevention tool against infectious diseases	222 (94.5)	39 (55.7)	2.2	0.5	10.4	4.8–22.6
Vaccinated against COVID-19	222/234 (94.9)	54 (77.1)				

Stepwise backward method was used. Variables eliminated when p-value >0,10. Model fit statistics: Χ^2^ (1) = 20.2 p-value <0.001; pseudo-R^2^ by Nagelkerke = 0.2; AUC (ROC): 0.7.

β: β coefficient, CI: confidence interval, MSM: men having sex with men, OR: odds ratio, SE: standard error for β coefficient.

Multivariable analysis showed that the belief in vaccination as a good tool against infectious diseases was the main predictor of general vaccine acceptance (Table 3).

## 4 Discussion

In this study, we assessed self-reported awareness regarding herpes zoster, its complications and its vaccination as well as (herpes zoster) vaccine acceptability among PLWH attending an outpatient clinic.

We found low awareness regarding herpes zoster, its complications and its link with varicella. Acceptance of the herpes zoster vaccine was more related to general attitudes toward vaccination than to knowledge about herpes zoster itself. It is worth noting that the vaccine in Belgium was recommended and reimbursed after the study period, so patients were likely unaware of its availability (Vaccination contre l'herpès zoster. Conseil Supérieur de la Santé, 2025; Remboursement. CBIP, 2025). Previous studies conducted in distinct cultural and medical settings reported higher proportion of herpes zoster awareness (Del Signore et al., 2020; Di Giuseppe et al., 2023; Roh et al., 2015; AlMuammar et al., 2023; Alhothali et al., 2023) as compared to our data, but most individuals from these studies also lacked knowledge regarding disease severity or its complications (Del Signore et al., 2020; Di Giuseppe et al., 2023; Roh et al., 2015; Al-Khalidi et al., 2022). Similarly, a low proportion of participants had knowledge of herpes zoster vaccine (ranging from 10.1 % to 55.8 %); our results are comparable to those from European studies involving patients without HIV infection (Del Signore et al., 2020; Di Giuseppe et al., 2023; Roh et al., 2015; AlMuammar et al., 2023; Alhothali et al., 2023; Al-Khalidi et al., 2022).

We identified one factor associated with herpes zoster vaccine acceptance: willingness to be vaccinated (general vaccination). A study among elderly found that general vaccine acceptance had the strongest positive association on herpes zoster vaccine acceptance, in addition to male sex and people believing that vaccination is an effective prevention tool (Del Signore et al., 2020). The need for additional information about herpes zoster vaccination was positively associated with herpes zoster vaccine acceptance in an Italian study (Di Giuseppe et al., 2023). While our data did not highlight the role of the patient's relatives, other studies showed that having a close relative with herpes zoster is positively associated with herpes zoster vaccine acceptance (Valente et al., 2016; Harpaz and Leung, 2017). Most female participants in our survey reported not having completed graduate studies (data not shown). The level of education among PLWH is linked to general vaccine coverage (Drewes et al., 2021; Tsachouridou et al., 2019). Also, higher education may have a positive influence on the willingness to get vaccinated against herpes zoster (Valente et al., 2016)*.* Migrant populations may less likely be vaccinated due to lower health literacy, cultural norms, and reduced access to healthcare (Larson et al., 2014; Daniels et al., 2022)*.* However, our results did not identify these factors as predictive, possibly because our study is monocentric, involves a smaller sample size and differs in sociocultural norms when compared to the studies cited.

The main reason for vaccine refusal was fear of side effects. Vaccine safety was also reported as a major hurdle to COVID-19 vaccine among PLWH in a study in France (Vallée et al., 2021). PLWH should be reassured that a numerous literature and pharmacovigilance data indicate that RZV is safe (Dauby et al., 2023). Our study also highlights poor knowledge of herpes zoster among PLWH. An interventional study among patients with chronic obstructive pulmonary disease demonstrated that informational video about herpes zoster vaccination had a positive impact on vaccine acceptance; such a tool should be evaluated among PLWH, and especially women (Yawn et al., 2022). Furthermore, doctor's recommendation (general practitioner) is a well-known factor increasing both herpes zoster knowledge and its vaccine acceptance (Bricout et al., 2019). General practitioners and HIV specialists should discuss specific vaccinations with PLWH.

One of the strengths of our study is the substantial representation of women of Sub-Saharan African ancestry, a population known to have higher rates of vaccine hesitancy (Abba-Aji et al., 2022). However, selection bias are likely, as participation was voluntary and most responders were French-speaking. Due to the cross-sectional design of the study, causal relationships cannot be established, and despite multivariable adjustments, the possibility of residual confounding from unmeasured variables cannot be excluded.

## 5 Conclusion

Herpes zoster vaccine acceptance in PLWH was moderate and was mostly related to general vaccination acceptance. Although most participants reported being aware of herpes zoster, their knowledge about the disease was limited. General vaccination acceptance may be positively influenced by the favorable opinion on the role of vaccination preventing infectious diseases. Given the aging PLWH population and the increased incidence of zoster with age, strategies to increase both knowledge and acceptance of herpes zoster vaccine should be developed.

## CRediT authorship contribution statement

**Christian Motet:** Writing – original draft, Methodology, Formal analysis, Conceptualization. **Agnès Libois:** Writing – review & editing, Conceptualization. **Charlotte Martin:** Writing – review & editing, Conceptualization. **Nicolas Dauby:** Writing – review & editing, Supervision, Methodology, Conceptualization.

## Declaration of competing interest

The authors declare that they have no known competing financial interests or personal relationships that could have appeared to influence the work reported in this paper.

## Data Availability

Data will be made available on request.

## Appendix A Supplementary data

Supplementary materiel should contain :- Part A is the survey that patients filled. It is attached to this article only for information. Part B contains supplementary data. I would rephrase the title of this subsection as "**Appendix and supplementary data**". Image 1
