# Supplementary material for: Awareness and acceptability of herpes zoster vaccination in people living with HIV
Source: Prev Med Rep. 2025 Jun 16;56:103143. doi: 10.1016/j.pmedr.2025.103143 (PMC12219356; doi:10.1016/j.pmedr.2025.103143)
Supplement: Supplementary file 1 — Supplementary materiel should contain :- Part A is the survey that patients filled. It is attached to this article only for information. Part B contains supplementary data. I would rephrase the title of this subsection as "Appendix and supplementary data". [file mmc1.docx]

# Appendix

# Survey: *Herpes Zoster Vaccination: Perception and Acceptance among People living with HIV*

Patient Inclusion code: …………………

1. Your maximum teaching level:

- Unschooled
- Primary school
- High school
- Graduate studies

Knowledge about Shingles/Herpes Zoster and the Shingle vaccine

1. Are you subject to any chronic disease(s)?

- Yes ○ No

1. If yes, what type(s)? (Several answers possible)

- Diabetes
- Cardiovascular disease(s)
- Pulmonary disease(s)
- Renal disease(s)
- Cancer

1. Do you know shingles?

- Yes ○ No

1. Did you know that the shingles virus and chickenpox are related?

- Yes ○ No

1. Have you ever had chickenpox?

- Yes ○ No ○ I don’t know

1. In your opinion, is shingles a severe disease?

- Yes ○ No ○ I don’t know

1. Do you think shingles can cause pain?

- Yes ○ No ○ I don’t know

1. If so, the pain is:

- In the short term
- In the long term
- In the short and long term
- I don’t know

1. Did you know there is a vaccine for shingles?

- Yes ○ No

1. If yes, what was your source of information? (Several answers possible)

- Family doctor/general practitioner
- Media
- Family/relatives
- Social networks
- Other: ……………….

1. Would you be willing to be vaccinated against shingles if you had risk factors for developing the disease?

- Yes ○ No ○ I don’t know

1. If no/if you don’t know, why? (Several answers possible)

- Fear of local effects
- Fear of long-term effects
- Vaccination is not mandatory
- The price
- Other: ……………….

1. In your opinion, how can shingles be prevented/avoided? (Several answers possible)

- Vaccination
- Hand hygiene
- Avoiding contact
- Other: ……………….

1. Do you think vaccination is the most effective way to prevent shingles?

- Yes ○ No ○ I don’t know

Personal Story of Shingles

1. Have you ever had shingles?

- Once
- More than once
- Never
- I don’t know

1. If yes, did you receive antiviral treatment for shingles?

- Yes ○ No ○ I don’t know

1. Did you feel the pain at the time of the rash?

- Yes ○ No ○ I don’t know

1. Did you experience pain after the rash disappeared (long-term pain)?

- Yes ○ No ○ I don’t know

1. Have you received treatment for these long-term pains?

- Yes ○ No ○ I don’t know

1. Has long-term pain limited your activities?

- Yes ○ No ○ I don’t know

1. If so, how much?

- A little
- Moderately
- A lot

1. Do you know someone who has had shingles?

- Yes ○ No

Perception of vaccination and vaccination coverage

1. Are you for or against vaccination in general?

- Absolutely for
- For
- Divided
- Against
- Totally against

1. Would you be willing to be vaccinated?

- Yes ○ No ○ I don’t know

1. Do you think that vaccination is a good method for the prevention of infectious diseases?

- Yes ○ No ○ I don’t know

1. Are you vaccinated against COVID-19 (coronavirus)?

- Yes
- No
- I don’t know
- I do not wish to answer

1. Your comments:

# Appendix

# Impact of age on herpes zoster vaccine acceptance in people living with HIV, CHU Saint-Pierre outpatient HIV center, Brussels-Belgium (2022-2023)


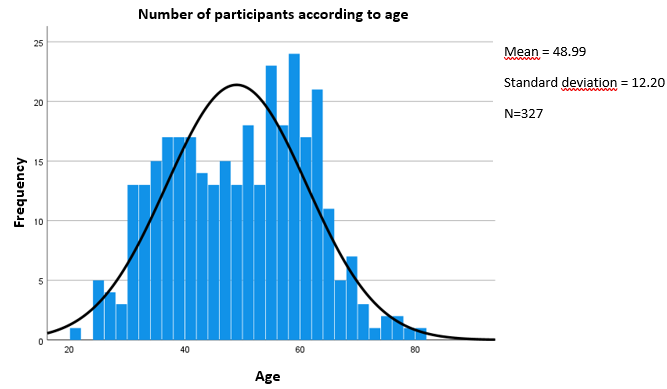
We can suggest that the distribution of the individuals based on age follows a normal distribution based on the above graphic, with a mean age of 48,99 (+/-12,20).

In Belgium, the vaccine is recommended from 60 years in immunocompetent individuals. Other European or American countries recommend recombinant zoster vaccine from 50 years or 65 years (Parikh et al. Expert Review of Vaccines. 2021.).

When performing univariate and multivariate analysis, age is considered as a continuous variable (Table 2) . Choosing a threshold at 50y, 60y or 65y did not statistically differ in the attitude towards herpes zoster vaccine acceptance (Table S1).

| Table S1. Age comparison according to herpes zoster vaccine acceptance | | | |
| --- | --- | --- | --- |
| Age group | Agree | Do not agree/do not know | p-value |
|  | n=201 | n=126 |  |
| < 50 years | 102 (50.7) | 58 (46.0) | 0.237 |
| < 60 years | 162 (80.6) | 94 (74.6) | 0.127 |
| < 65 years | 185 (92.0) | 113 (89.7) | 0.295 |
